# The impact of SARS-CoV-2 infection and vaccination on inflammatory arthritis: a cohort study

**DOI:** 10.3389/fimmu.2023.1207015

**Published:** 2023-07-26

**Authors:** Giovanni Striani, Ariela Hoxha, Mariagrazia Lorenzin, Giacomo Cozzi, Laura Scagnellato, Tatiana Vangelista, Francesca Frizzera, Pierino De Sandre, Paolo Simioni, Andrea Doria, Roberta Ramonda

**Affiliations:** ^1^ Rheumatology Unit, Department of Medicine (DIMED), University of Padua, Padua, Italy; ^2^ General Internal Medicine and Thrombotic and Hemorrhagic Unit, Department of Medicine (DIMED), University of Padua, Padua, Italy; ^3^ Occupational Medicine Service, Treviso, Italy; ^4^ Internal Medicine Unit, Department of Medicine, San Bortolo Hospital, Vicenza, Italy

**Keywords:** rheumatoid arthritis, ankylosing spondylitis, psoriatic arthritis, COVID-19, SARS-CoV-2, vaccines, treatment

## Abstract

**Objectives:**

To investigate the effects of SARS-CoV-2 infection, as well as short- (within 48 hours) and long-term (within 30 days) adverse events (AEs) of SARS-CoV-2 vaccines, including arthritis flares in a large cohort of patients with inflammatory arthritis (IA).

**Methods:**

A retrospective cohort study comprising 362 patients: 94 (26%) rheumatoid arthritis, 158 (43.6%) psoriatic arthritis and 110 (30.4%) ankylosing spondylitis; and 165 healthy controls (HC) to ascertain the prevalence and severity of SARS-CoV-2 infection in patients with IA, the rate of AEs associated with SARS-CoV-2 vaccines and disease flares within a month of the vaccination. All patients provided informed consent and data about SARS-CoV-2 infection and/or vaccination status.

**Results:**

One-hundred-seventeen (32.3%) patients and 39 (23.6%) HC were affected by SARS-CoV-2 infection. Forty (34.2%) patients experienced an IA flare within one month of infection, of whom 3 (7.5%) needed to switch therapy. The prevalence of SARS-CoV-2 infection, disease severity, and hospitalization rate were not significantly different. At least one shot of SARS-CoV-2 vaccine was administered in 331 (91.4%) patients and 147 (89.1%) HC. Within 48 hours, 102 (30.8%) patients developed vaccine-related AEs; 52 (15.7%) patients with >1 vaccine dose experienced an IA flare-up, of whom 12 (23.1%) needed to switch therapy.

**Conclusions:**

A significantly higher rate of IA flare was observed among patients who contracted SARS-CoV-2 infection vs. those without infection. Patients with IA experienced flares after SARS-CoV-2 vaccination, though it was not statistically significant.

## Highlights

- The prevalence of SARS-CoV-2 infection, COVID-19 severity, and the rate of hospitalization were not significantly different in our inflammatory arthritis patients compared with the healthy controls.
- A significantly higher flare rate of inflammatory arthritis was observed among patients who contracted SARS-CoV-2 infection compared to those without infection.
- Vaccination was not associated with flare-ups of inflammatory arthritis in our cohort.
- Female sex emerged as the only predisposing risk factor for adverse events following immunization.

## Introduction

Since the beginning of the severe acute respiratory coronavirus 2 (SARS-CoV-2) pandemic in late 2019, there has been growing concern among physicians and patients dealing with inflammatory arthritis (IA), regarding the risk of exacerbating joint diseases or developing more severe clinical manifestations of Coronavirus disease 2019 (COVID-19) than the general population. Most of these patients are undergoing immunosuppressant therapy — mainly anti-cytokine therapy. Although this may result in a greater risk of developing severe infections, many cytokines such as interleukin (IL) 1, IL-6 and Tumor Necrosis Factor-alpha (TNFα) are involved in the cytokine storm that determines the severity of COVID-19 (1).

Several studies have indicated a similar or slightly increased severity of COVID-19 and risk of hospitalization in patients with inflammatory joint disease, and more widely in rheumatic diseases, vs. the general population (2–8). There was no association between SARS-CoV-2 infection and joint disease exacerbation (9, 10). The serological response in patients with IA was surprisingly higher than expected based on reported symptoms (11), despite immunosuppressive therapy. However, some data suggests that patients with rheumatoid arthritis (RA) appear to have a higher risk of contracting SARS-CoV-2 infection (12), developing more severe COVID-19 (13) and joint disease exacerbation following infection (14), and need for therapeutic switch (15), vs. patients with seronegative spondyloarthritis (SpA).

The launch of the COVID-19 vaccination campaign in late 2020 raised some concerns among patients and physicians about the risk of developing adverse events following immunization (AEFI) and joint disease exacerbation following the vaccination. Several studies have found that patients with IA, or rheumatic diseases in general, do not carry a higher risk (16–19) than the general population. Although joint disease flare-ups following vaccination appear to be very rare (20–22) or completely absent in some cohorts (23, 24), some studies conducted on larger populations found a slightly higher incidence of exacerbation than previously reported, estimated at <20% (25, 26). These flare-ups are generally mild and easily manageable with therapy (27). Predisposing factors include the use of corticosteroids, a history of other autoimmune diseases and the presence of a previous exacerbation over the past 12 months (28).

The latest European Alliance of Associations for Rheumatology (EULAR) recommendations (29) and the American College of Rheumatology (ACR) recommendations (30) concur on the importance of the COVID-19 vaccine in patients with rheumatic diseases, stressing that there is a theoretical risk of a joint disease flare-up albeit much lower than the benefit conferred by immunization during a pandemic.

Thus, we designed this study to evaluate the impact of SARS-CoV-2 infection and/or vaccination in a cohort of patients with IA in Northeast Italy.

## Materials and methods

### Design, setting, and study population

We conducted a retrospective cohort study and enrolled all consecutive patients with IA who attended the Spondyloarthritis Clinic at Padova University Hospital and Arthritis Clinic-San Bortolo Hospital (Vicenza), as well as healthy controls attending the Occupational Medicine Clinic for routine health surveillance activities between May 2020 and May 2022. Patients with IA and a confirmed diagnosis of ankylosing spondylitis (AS) according to the modified New York criteria (31), psoriatic arthritis (PsA) according to CASPAR criteria (32), and with rheumatoid arthritis (RA) according to the ACR criteria (33) were included. Exclusion criteria were: *i*) patients under the age of 18 and *ii*) patients unable or who refused to provide written informed consent. No patients fulfilled the exclusion criteria.

All enrolled patients provided written informed consent, in accordance with the principles of the Declaration of Helsinki. Each participating Centre received the approval of the local Ethics Committee [approval no. CESC code: 4930/AO/2. URC: AOP2073], as well as the written informed consent for the anonymous use of personal data from every patient, in compliance with Italian Legislative Decree 196/2003.

### Outcome measures

Demographics and clinical data were collected for all participants, including age, type of rheumatic disease, ongoing medications, and comorbidities. The presence of concomitant therapies and comorbidities were investigated (yes/no) during a face-to-face interview at one of the scheduled assessment visits, and by reviewing the patient’s medical records. Comorbidities such as metabolic diseases (diabetes, obesity), vascular diseases (hypertension, coronary heart disease, cerebrovascular disease), neoplastic diseases (solid and hematological), pulmonary diseases (asthma, chronic obstructive pulmonary disease, pulmonary fibrosis) were recorded. Treatments with *biological disease-modifying anti-rheumatic drugs* bDMARDs (anti-TNFα, anti-IL17A, anti-IL12/23p40, anti-IL6, CTLA-4Ig and anti-CD20), *conventional disease-modifying anti-rheumatic drugs* csDMARDs (methotrexate, leflunomide, sulfasalazine, hydroxychloroquine), *targeted synthetic disease-modifying anti-rheumatic drugs* tsDMARDs such as phosphodiesterase 4 inhibitor and Janus-activating kinases (JAK) inhibitors (tofacitinib, upadacitinib, baricitinib, filgotinib) or corticosteroid therapy were also recorded.

Disease activity was assessed by Ankylosing Spondylitis Disease Activity Score (ASDAS-CRP) (34) for AS and by Disease Activity Score (DAS28-CRP) (35, 36) for PsA and RA. Based on this score the disease activity was classified as remission, low disease activity (LDA), and high disease activity, respectively when ASDAS CRP < 1.3 or DAS28CRP <2.6, ASDAS CRP > 1.3 and < 2.1 or DAS28CRP >2.6 and <3.2, ASDAS CRP >2.1 or DAS28CRP >3.2 were registered.

All disease flares were documented in medical reports, laboratory evaluations, describing symptoms, activity disease score, and the patient’s clinical history. All patients were evaluated during telemedicine visits and those who needed to switch therapy were evaluated in face-to-face visits.

Information on SARS-CoV-2 infection, and on vaccination, were also collected by an interview conducted as shown in **Supplementary Table**.

Side effects were assessed in accordance with the World Health Organization (WHO) guidelines on AEFI (37, 38). SARS-CoV-2 infection was considered only if documented in accordance with current laws in Italy, first only *via* nasopharyngeal swab for molecular tests, and later *via* nasopharyngeal swab for rapid antigen tests. The severity of COVID-19 was assessed as indicated by the National Institute of Health (NIH): “asymptomatic or presymptomatic infection [no symptoms that are consistent with COVID-19]; mild illness [any of the various signs and symptoms of COVID-19, e.g., fever, cough, sore throat, malaise, headache, muscle pain, nausea, vomiting, diarrhea, loss of taste and smell but not shortness of breath, dyspnea, or abnormal chest imaging]; moderate illness [evidence of lower respiratory disease during clinical assessment or imaging and oxygen saturation measured by pulse oximetry (SpO2) ≥94% on room air at sea level]; severe illness [SpO2 <94% on room air at sea level, ratio of arterial partial pressure of oxygen to fraction of inspired oxygen (PaO2/FiO2) <300 mm Hg, respiratory rate >30 breaths/min, or lung infiltrates >50%], critical illness [respiratory failure, septic shock, and/or multiple organ dysfunction]” (39). Data regarding SARS-CoV-2 infection or vaccination status were compared with controls.

The primary endpoint was the presence of joint disease flare-ups following SARS-CoV-2 infection or vaccination, by comparing disease activity indices before and after infection and/or vaccination. The secondary endpoints were: *i*) the identification of possible predictive factors of flare-ups such as age, gender, comorbidity, baseline disease activity grade, or class of anti-rheumatic drugs; *ii*) the risk of flare-ups between the two different clinical entities considered in the study (seronegative spondyloarthritis and rheumatoid arthritis) after infection and/or vaccination; *iii*) the incidence of SARS-CoV-2 infection, the severity of COVID-19, and any AEFI in cases and controls.

### Statistical analysis

Data distribution (normal or not normal) was verified through graphical representation and then verified using the Shapiro-Wilk normality test. The data were expressed as mean (standard deviation) in case of normal distribution, and median (interquartile range –IQR) in case of non-normal distribution, for continuous variables. Categorical variables were expressed as numbers (percentages). Baseline characteristics across the 3 groups (AS, PsA, and RA) were compared through the Mann-Whitney test for independent samples in the case of continuous variables and Chi-square (χ2) for categorical variables. Comparison between 2 groups (patients and controls) were performed using Wilcoxon rank sum/signed rank tests (as most data were not normally distributed) for continuous variables, and χ2 test for categorical variables, as appropriate. A logistic regression analysis was carried out to identify predictors of disease flare-ups. The following covariates were examined: age, sex, comorbidity, baseline disease activity grade, class of anti-rheumatic drugs and SARS-CoV-2 infection.

All statistical analyses were carried out using GraphPAD, PRISM9program and SPSS 27.0 statistical software; p values < 0.05 were considered as significant.

## Results

The demographic and baseline characteristics of the study cohort are highlighted in **Table 1**. Between May 2020 and May 2022, we enrolled a total of 362 patients, 182 (50.3%) females and 180 (49.7%) males with a median age of 57 years, IQR 47-66, and 165 HC, 22 (13.3%) females and 143 (86.7%) males with a median age of 45 years, IQR 34-52. Sex distribution between the two groups and age at enrollment were statistically significant (p <0.0001 for both).

**Table 1:** Demographic and baseline features of the study cohort.

	Study cohort (n=362)
Female, n (%)	182 (50.3)
Median age, years (IQR)	57 (47-66)
Disease	
Rheumatoid Arthritis, n (%)	94 (26.0)
Psoriatic Arthritis, n (%)	158 (43.6)
Ankylosing Spondylitis, n (%)	110 (30.4)
Therapy	
Steroids, prednisone 5-15 mg/day, n (%)	41 (11.3)
cDMARDs	
Methotrexate, n (%)	91 (25.1)
Leflunomide, n (%)	22 (6.1)
Sulfasalazine, n (%)	16 (4.4)
Hydroxychloroquine, n (%)	8 (2.2)
bDMARDs	
Anti-TNFɑ	197 (54.4)
Anti-CD20	7 (1.9)
Abatacept	16 (4.4)
Anti-IL-6	14 (3.9)
Anti-IL-17	65 (17.9)
Anti-IL-23	8 (2.2)
JAK-i	13 (3.6)
Apremilast	8 (2.2)
Disease Activity ^	
Active Disease, n (%)	81 (22.4)
Low Disease Activity, n (%)	58 (16.0)
Remission, n (%)	223 (61.6)
Comorbidities	
Cardiovascular disease, n (%)	115 (33.5)
Diabetes, n (%)	28 (12.7)
Obesity, n (%) *	43 (18.9)
Pulmonary disease, n (%)	17 (4.9)
Cancer, n (%)	17 (4.9)

IQR, interquartile range; cDMARDs, conventional disease-modifying antirheumatic drugs; bDMARDs, biological disease-modifying antirheumatic drugs; TNFɑ , tumor necrosis factor ɑ; CD20, cluster of differentiation 20 (B-lymphocyte antigen); IL, interleukin; JAK-i, Janus kinase inhibitors.

^^^Disease activity evaluated by ASDAS-CRP (Ankylosing Spondylitis Disease Activity Score-C Reactive protein) and DAS28-CRP (Disease Activity Score-C Reactive Protein), define as remission (ASDAS-CRP <1.3, DAS28-CRP <2.6), low disease activity (ASDAS-CRP 1.3-2.0, DAS28-CRP 2.6-3.2), active disease (ASDAS-CRP ≥2.1, DAS28-CRP ≥3.2).

*Obesity is evaluated by BMI (Body Mass Index), defined as BMI >30.0.

Among 362 patients with IA, 94 (26.0%) patients were affected by RA, 158 (43.6%) PsA, and 110 (30.4%) AS. Forty-one (11.3%) patients received steroid therapy with an equivalent dose of prednisone 5-15 mg daily. Methotrexate was the most frequently prescribed cDMARD in 91 (25.1%) patients, whereas anti-TNFα was the most frequently prescribed bDMARD in 197 (54.4%) patients. Regarding comorbidities, cases had a statistically significant prevalence of cardiovascular diseases as well as obesity and diabetes (p<0.0001 for all), whereas there was no difference in pulmonary diseases and cancer vs. healthy controls.

### Impact of SARS-CoV-2 infection on inflammatory arthritis

Clinical characteristics relating to SARS-CoV-2 infection in our study population are reported in **Table 2**. One hundred-seventeen (32.3%) patients and 39 (23.6%) controls contracted a SARS-CoV-2 infection during the study period. However, the infection rate was not statistically significant (p=0.05 OR 1.5, 95% CI: 1.0 to 2.3). Mild infection was the most frequent clinical presentation of SARS-CoV-2 infection: 81.2% of patients vs. 92.1% of controls). Hospitalization rate was higher among cases than healthy controls (9.4% vs. 5.1) though not statistically significant. One death was recorded in our study cohort.

**Table 2:** Prevalence and severity of SARS-CoV-2 infection in inflammatory arthritis.

	Inflammatory arthritis	Healthy control	p-value	OD (95%CI)
**SARS-CoV-2 rate, n (%)**	117 (32.3)	39 (23.6)	0.05	1.54 (1.01-2.34)
SARS-CoV-2 severity *				
Asymptomatic, n (%)	9 (7.7)	1 (2.6)		
Mild, n (%)	95 (81.2)	35 (92.1)		
Moderate, n (%)	6 (5.1)	0 (0)	ns	
Severe, n (%)	2 (1.7)	1 (2.6)		
Critical, n (%)	5 (4.3)	1 (2.6)		
**Hospitalization rate, n (%)**	11 (9.4)	2 (5.1)	ns	
**Death, n (%)**	1 (0.9)	0 (0)	ns	

SARS-CoV-2, severe acute respiratory syndrome coronavirus 2.

*SARS-CoV-2 severity in healthy controls calculated from 38 subjects.

ns, not statistical significant.

**Figure 1:**
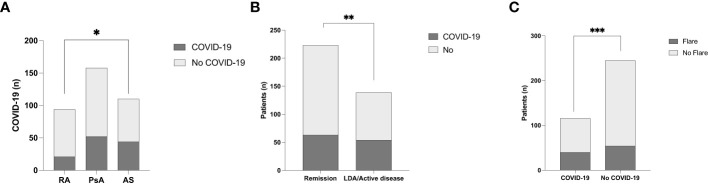
**(A)** SARS-CoV-2 infection rate in the different inflammatory arthritis subgroups. *p<0.01. **(B)** COVID-19 rate according to disease activity of inflammatory arthritis. **p=0.04. **(C)** The prevalence of inflammatory disease flares in relation to SARS-CoV-2 infection ***p=0.03.

One-hundred-seventeen (32.3%) patients were affected by SARS-CoV-2 infection. The prevalence of SARS-CoV-2 infection was significantly different between the IA subgroups, (p=0.03); in particular patients with AS had significantly higher infection rates vs. RA (p=0.01), as shown in **Table 3** and **Figure 1**.

**Table 3:** Clinical features of inflammatory arthritis according to SARS-CoV-2 infection status.

	Total population (n=362)	SARS-CoV-2 infection (n=117)	No SARS-CoV-2 infection (n=245)	p-value
Female, n (%)	182 (50.3)	62 (53.0)	120 (49.0)	ns
Median age, years (IQR)	57 (47-66)	55 (44-62)	58 (48-68)	0.02
Disease				
Rheumatoid Arthritis, n (%)	94 (26.0)	21 (18.0)	73 (29.8)	
Psoriatic Arthritis, n (%)	158 (43.6)	52 (44.4)	106 (43.3)	0.03
Ankylosing Spondylitis, n (%)	110 (30.4)	44 (37.6)	66 (26.9)	
Therapy				
Steroids§, n (%)	41 (12.0)	10 (24.4)	31 (75.6)	ns
cDMARDs				
Methotrexate, n (%)	91 (25.1)	25 (21.4)	66 (26.9)	
Leflunomide, n (%)	22 (6.1)	5 (4.3)	17 (6.9)	ns
Sulfasalazine, n (%)	16 (4.4)	7 (6.0)	9 (3.7)	
Hydroxychloroquine, n (%)	8 (2.2)	1 (0.9)	7 (2.9)	
bDMARDs				
Anti-TNFɑ	210 (54.4)	67 (57.3)	143 (58.4)	
Anti-CD20	7 (1.9)	2 (1.7)	5 (2.0)	
Abatacept	16 (4.4)	4 (3.4)	12 (4.9)	
Anti-IL-6	14 (3.9)	1 (0.8)	13 (5.3)	ns
Anti-IL-17	65 (18.0)	25 (21.4)	40 (16.3)	
Anti-IL-23	8 (2.2)	5 (4.3)	8 (3.3)	
JAK-i	13 (3.6)	4 (3.4)	9 (3.7)	
Apremilast	7 (1.9)	0 (0)	7 (2.8)	
Disease Activity ^				
LDA/Active Disease, n (%)	139 (38.4)	54 (46.2)	85 (34.7)	0.04
Remission, n (%)	223 (61.6)	63 (53.8)	160 (65.3)	
Comorbidities				
Cardiovascular disease	115 (31.8)	35 (29.9)	80 (32.6)	
Diabetes	28 (7.7)	8 (6.8)	20 (8.2)	
Obesity *	43 (11.9)	15 (12.8)	28 (11.4)	ns
Pulmonary disease	17 (4.7)	10 (8.5)	7 (2.8)	
Cancer	17 (4.7)	9 (7.7)	8 (3.3)	

IQR, interquartile range; cDMARDs, conventional disease-modifying antirheumatic drugs; bDMARDs, biological disease-modifying antirheumatic drugs; TNFɑ , tumor necrosis factor ɑ ; CD20, cluster of differentiation 20 (B-lymphocyte antigen); IL, interleukin; JAK-i, Janus kinase inhibitors; LDA, low disease activity; SARS-CoV-2, severe acute respiratory syndrome coronavirus 2.

§ prednisone 5-15 mg/day.

^ Disease activity evaluated by ASDAS-CRP (Ankylosing Spondylitis Disease Activity Score-C Reactive protein) and DAS28-CRP (Disease Activity Score-C Reactive Protein), define as remission (ASDAS-CRP <1.3, DAS28-CRP <2.6), low disease activity (ASDAS-CRP 1.3-2.0, DAS28-CRP 2.6-3.2), active disease (ASDAS-CRP ≥2.1, DAS28-CRP ≥3.2).

* Obesity is evaluated by BMI (Body Mass Index), defined as BMI >30.0.

ns, not statistical significant.

Sixty-three (53.8%) of patients with IA in remission contracted a SARS-CoV-2 infection vs. 54 (46.2%) patients with active/low disease activity, as shown in **Table 3**. The prevalence of COVID-19 was significantly higher in patients in remission vs. those with high/low disease activity (p=0.04 OR 0.62, 95% CI: 0.39 to 0.97) as shown in **Figure 1**. Forty patients (34.2%) experienced a flare within one month of COVID-19. A significantly higher rate of flare-ups was observed among patients who contracted a SARS-CoV-2 infection (p=0.01 OR 1.86, 95% CI: 1.16 to 3.05) vs. those without infection, as shown in **Figure 1**. The median (IQR) ASDAS PCR and DAS28 PCR during flares were 2.8 (2.4-3.7) and 3.5 (3.0-4.1), respectively — significantly higher vs. before flares (p<0.001 for both). The need to switch to another therapy or initiate NSAIDs occurred in 3/40 (7.5%) and 13 (32.5%) patients, respectively.

We found no significant differences in SARS-CoV-2 infection rate and disease severity between RA, PsA, and AS. Moreover, there was no significant difference in terms of SARS-CoV-2 infection rate and severity according to disease activity and the different therapies.

### Impact of SARS-CoV-2 vaccination on inflammatory arthritis

The clinical characteristics of patients with IA and vaccination status are reported in **Table 4**. Three-hundred-thirty-one (91.4%) patients received at least one dose of a SARS-CoV-2 vaccine. There was no difference in the SARS-CoV-2 vaccination rate between cases and healthy controls, as well as between the different subsets of IA. No difference was observed regarding the different therapeutic regimens and the comorbidities between vaccinated patients vs. unvaccinated. Most patients (83.2%) received the BTN162b2 (BioNTech/Pfizer, Mainz Germany) vaccine. In our cohort 65.5%, 32.3%, and 2.1% of the patients received three, two, and one dose of the vaccine, respectively. Among healthy controls, 91.8% and 8.2% received two and one dose, respectively. Fear was the most frequently recorded reason for not getting vaccinated in 18/31 (58.1%). The rate of unvaccinated was similar between males and females (48.4% vs. 51.6%) as was the most frequent reason for not getting vaccinated: fear (60% vs. 56.3%).

**Table 4:** Clinical features of inflammatory arthritis patients according to the vaccination status.

	Total population (n=362)	Vaccinated (n=331)	Unvaccinated (n=31)	p-value
Female, n (%)	182 (50.3)	166 (50.2)	16 (51.6)	ns
Median age, years (IQR)	57 (47-66)	57 (47-66)	60 (48-63)	ns
Disease				
Rheumatoid Arthritis, n (%)	94 (26.0)	82 (24.8)	12 (38.7)	
Psoriatic Arthritis, n (%)	158 (43.6)	144 (43.5)	14 (45.2)	ns
Ankylosing Spondylitis, n (%)	110 (30.4)	105 (31.7)	5 (16.1)	
Therapy				
Steroids§, n (%)	41 (12.0)	41 (12.4)	2 (14.3)	ns
cDMARDs				
Methotrexate, n (%)	91 (25.1)	83 (25.1)	8 (25.8)	
Leflunomide, n (%)	22 (6.1)	22 (6.6)	0 (0)	ns
Sulfasalazine, n (%)	16 (4.4)	16 (4.8)	0 (0)	
Hydroxychloroquine, n (%)	8 (2.2)	7 (2.1)	1 (3.2)	
bDMARDs				
Anti-TNFɑ	210 (54.4)	190 (57.4)	20 (64.5)	
Anti-CD20	7 (1.9)	7 (2.1)	0 (0)	
Abatacept	16 (4.4)	12 (3.6)	4 (12.9)	
Anti-IL-6	14 (3.9)	12 (3.6)	2 (6.5)	ns
Anti-IL-17	65 (18.0)	62 (18.7)	3 (9.7)	
Anti-IL-23	8 (2.2)	8 (2.4)	0 (0)	
JAK-i	13 (3.6)	12 (3.6)	1 (3.2)	
Apremilast	8 (1.9)	8 (2.4)	0 (0)	
Disease activity ^				
LDA/Active Disease, n (%)	139 (38.4)	129 (39.0)	10 (32.3)	ns
Remission, n (%)	223 (61.6)	202 (61.0)	21 (67.7)	
Comorbidities				
Cardiovascular disease	115 (31.8)	106 (32.0)	9 (29.0)	
Diabetes	28 (7.7)	26 (7.9)	2 (6.5)	
Obesity *	43 (11.9)	41 (12.4)	2 (6.5)	ns
Pulmonary disease	17 (4.7)	16 (4.8)	1 (3.2)	
Cancer	17 (4.7)	17 (5.1)	0 (0)	
Vaccine type °				
Comirnaty (BioNTech, Pfizer)		273 (83.2)	–	
Spikevax (Moderna)		28 (8.6)	–	
Vaxzevria (Oxford/Astrazeneca)		27 (8.2)	–	
Vaccine shots °				
3 shots		215 (65.6)	–	
2 shots		106 (32.3)	–	
1 shot		7 (2.1)	–	
Reason for missed vaccination, n (%)				
Total 31 (8.6)				
Fear		–	18 (58.1)	
Medical contraindication		–	7 (22.6)	
Other		–	6 (19.3)	
Males		–		
Fear		–	9 (60.0)	
Medical contraindication		–	3 (20.0)	
Other		–	3 (20.0)	
Females		–		
Fear		–	9 (56.3)	
Medical contraindication		–	4 (25.0)	
Other		–	3 (18.7)	

IQR, interquartile range; cDMARDs, conventional disease-modifying antirheumatic drugs; bDMARDs, biological disease-modifying antirheumatic drugs; TNFɑ , tumor necrosis factor ɑ ; CD20, cluster of differentiation 20 (B-lymphocyte antigen); IL, interleukin; JAK-i, Janus kinase inhibitors; LDA, low disease activity.

^ Disease activity evaluated by ASDAS-CRP (Ankylosing Spondylitis Disease Activity Score-C Reactive protein) and DAS28-CRP (Disease Activity Score-C Reactive Protein), define as remission (ASDAS-CRP <1.3, DAS28-CRP <2.6), low disease activity (ASDAS-CRP 1.3-2.0, DAS28-CRP 2.6-3.2), active disease (ASDAS-CRP ≥2.1, DAS28-CRP ≥3.2).

§ prednisone 5-15 mg/day.

* Obesity is evaluated by BMI (Body Mass Index), defined as BMI >30.0.

° Vaccine shots calculated from 328 patients.

AEFI in patients with IA are highlighted in **Table 5**. The prevalence of vaccine side effects was significantly higher in the control group vs. patients (44.9% vs. 31.2%, p=0.005 OR 0.55, 95% CI 0.37-0.82). Asthenia within 48 hours from the vaccination was the most frequently reported AE with 65 (63.7%) patients, followed by fever and arthralgia in 51 (50%) and 42 (41.2%) patients, respectively. Overall, females reported a significantly higher rate of AEs both within 48 hours and within 30 days from the vaccination (p=0.003 and p=0.004). However, there were no statistically significant differences as regards sex-related adverse events in our cohort. Moreover, we did not find any significant difference regarding adverse events according to disease activity status and various therapeutic regimens.

**Table 5:** Adverse events in inflammatory arthritis patients after anti-SARS-CoV-2 vaccination.

Total	AEs within 48 h (n=102)	p-value	Fever within 48 h (n=51)	p-value	Arthralgia within 48 h (n=43)	p-value	Asthenia within 48 h (n=65)	p-value	Disease flares within 30 days (n=52)	p-value	Other AEs within 30 days (n=35)	p-value
**Active vs Inactive disease ^**	43 (42.2) vs 59 (57.8)	0.46	18 (41.9) vs 33 (55.9)	0.23	19 (44.2) vs 24 (40.7)	0.84	26 (66.1) vs 39 (60.5)	0.68	23 (14.4) vs 29 (17.8)	0.44	15 (9.9) vs 20 (11.6)	0.71
**Steroids treatment§ + vs -**	15 (38.5) vs 87 (29.8)	0.27	8 (53.3) vs 43 (49.4)	0.99	5 (33.3) vs 38 (43.7)	0.58	11 (73.3) vs 54 (62.1)	0.56	6 (15.4) vs 46 (15.7)	0.99	7 (17.9) vs 28 (9.6)	0.16
cDMARDs + vs - *	36 (28.6) vs 66 (32.2)	0.54	19 (52.8) vs 32 (48.5)	0.84	17 (47.2) vs 26 (39.4)	0.53	24 (66.7) vs 41 (62.1)	0.67	18 (14.3) vs 34 (16.6)	0.64	12 (9.5) vs 23 (11.2)	0.71
**bDMARDs + vs - °**	95 (30.5) vs 7 (36.8)	0.61	49 (51.6) vs 2 (28.6)	0.44	37 (38.9) vs 6 (46.2)	0.76	63 (66.3) vs 2 (28.6)	0.09	48 (15.4) vs 4 (21.1)	0.52	33 (10.6) vs 2 (10.5)	0.99
**Female vs Male**	64 (38.6) vs 38 (23.0)	**0.003**	34 (53.1) vs 17 (44.7)	0.54	27 (42.2) vs 16 (42.1)	0.99	41 (64.1) vs 24 (63.2)	0.99	30 (18.1) vs 22 (13.3)	0.29	26 (15.7) vs 9 (5.5)	**0.004**

AE, adverse events; cDMARDs, conventional disease-modifying antirheumatic drugs; bDMARDs, biological disease-modifying antirheumatic drugs.

^ “Active disease” includes both LDA (low disease activity) and active disease, “inactive disease” includes only remission; disease activity evaluated by ASDAS-CRP (Ankylosing Spondylitis Disease Activity Score-C Reactive protein) and DAS28-CRP (Disease Activity Score-C Reactive Protein), define as remission (ASDAS-CRP <1.3, DAS28-CRP <2.6), low disease activity (ASDAS-CRP 1.3-2.0, DAS28-CRP 2.6-3.2), active disease (ASDAS-CRP ≥2.1, DAS28-CRP ≥3.2).

§ prednisone 5-15 mg/day.

* cDMARDs include methotrexate, leflunomide, sulfasalazine, hydroxychloroquine.

° bDMARDs include anti-TNFɑ , anti-CD20, abatacept, anti-IL-6, anti-IL-17, anti-IL-23, JAK-i, apremilast.

Bold values: statistical significant.

Fifty-two (15.7%) patients experienced a joint disease flare within one month of vaccination vs. 9/31 (29%) unvaccinated patients. However, the flare rate was not significantly different between vaccinated vs. unvaccinated patients. There was a higher rate of active disease in the last 12 months among patients who experienced flares vs. those who did not (30.8% vs. 20.1%), though the difference was not statistically significant. The median (IQR) ASDAS PCR and DAS28 PCR during flares were 3.2 (2.6-3.6) and 3.7 (3.1-4.6), respectively — significantly higher vs. before flares (p=0.003 and p=0.04, respectively). Twelve (23.1%) and 29 (55.8%) patients switched to another therapy or initiated NSAIDs, respectively.

In the multivariate analysis, we did not find any independent predictors of IA flares.

## Discussion

Overall, our study found a significantly higher risk of joint disease flare-ups within one month of SARS-CoV-2 infection. However, there was no difference in the rate of flare within the different IA subsets. At multivariate analysis, the only predictive risk factor for a flare-up was SARS-CoV-2 infection, thus confirming previous findings in the literature (9, 10). Only 7.5% of patients who experienced flares needed a therapy switch. The prevalence of SARS-CoV-2 infection was higher among cases than in the control group (32.3% *vs.* 23.6%), though not statistically significant. Furthermore, there was no significant difference as regards the risk of hospitalization and the more severe course of COVID-19 between cases and healthy controls. Only one death occurred during the study, a patient with several comorbidities and long-term RA. Some recent data has shown that RA patients have a higher risk of contracting SARS-CoV-2 infection (12) and developing more severe COVID-19 (13) than patients with seronegative spondyloarthritis. Surprisingly, we found a significantly higher prevalence of SARS-CoV-2 infection in AS than in RA patients, in younger ones, and in those in remission. The cytokine profile involved in the pathogenesis of lung damage in COVID-19 is similar to that observed in the pathogenesis of joint damage in RA, with IL1, IL6 and TNF-α as key players (40, 41). Instead, a central role in the pathogenesis of AS is attributed to IFN-γ, IL12, IL17, IL22, IL23 (42). Hence, the expectedly higher rate of infections in RA vs. SA group. Thus, we believe that the difference in the rate of infection between AS and RA group is due to the demographic differences between the two subgroups: patients with AS tend to be young adults, therefore much more exposed to social contacts that pose a risk of infection, as opposed to patients with RA who tend to be older and are therefore more likely to have limited their social contacts and followed the prevention rules more assiduously during the pandemic. The difference cannot be attributed either to different therapies administered to the two subgroups: patients with AS were treated mainly with anti-IL12, IL17, IL23, and anti-TNF-α whereas patients with RA were treated mostly with MTX, corticosteroids, anti-TNF-α, anti-IL6, JAKi, abatacept, and rituximab. Finally, we did not found any differences in incidence of flares between the various subgroups despite the pathogenesis described possibly suggesting a greater risk for patients with RA. The different prevalence of RA and seronegative SpA between our patients and the general population may be attributable to the fact that the Rheumatology clinic of Padova University Hospital is mainly dedicated to SpA. However, we did not find any difference regarding the risk of developing more severe COVID-19 and having a higher rate of joint disease flares between the IA subsets, and those with high disease activity. Furthermore, we fail to demonstrate any impact of the different anti-rheumatic drugs on SARS-CoV-2 infection and/or COVID-19 course, in particular as it pertains to three recently reported aspects: the potential protective role of TNFi (43, 44) and the negative effect of corticosteroids and rituximab, the more severe forms of COVID-19 in patients with immune-mediated rheumatic diseases (5, 43, 44), and sulfasalazine. The effect of TNFi may stem from the fact that most of our patients suffering from seronegative SpA which presents some pathophysiological differences vs. RA, such as T17/T1 pathway balance (10). We did not find a negative effect of RTX, SSZ or steroids, as previously reported (5, 43, 44), likely due to the low frequency of this treatment in our cohort.

Although our findings showed no association between the SARS-CoV-2 vaccine and the occurrence of joint disease flare-ups, the latter were easily manageable with NSAIDs, as widely described in literature (20–22, 27). It bears noting that 23% of the flares required a therapy switch in those patients with active disease in the last 12 months. Nonetheless, there was no difference in risk of flare within different IA subsets and therapy options. Unlike previous studies, we found no increased risk of flare in patients treated with steroids or immunosuppressive drugs, or in those who suffered a previous exacerbation in the past 12 months (28). Moreover, we observed no other predisposing risk factors such as age, sex, or comorbidities despite a higher rate of active disease in the last 12 months in patients who experienced flares vs. those who did not.

Finally, as widely described in the literature (18–21, 24), we observed no increased rate of AEFI than in the healthy controls. Female sex emerged as the only predisposing risk factor for AEFI both within 48 hours and 30 days despite no significant differences relating to disease activity status and different therapeutic regimens.

1. We would be remiss to not mention some of the limitations of our study. Firstly, the retrospective design may have resulted in recall bias. Secondly, our cases and controls were not matched for age and sex, though there were no significant differences in the rate of infection and AEFI between the two groups.

Overall, our findings did not show a more severe course of COVID-19 in patients with IA, despite a slightly higher rate of flare-ups. Moreover, COVID-19 vaccines were well tolerated and did not correlate with an increased risk of flares. Thus, vaccination is advisable in this subset of patients, especially considering their overall frailty.

## Data availability statement

The raw data supporting the conclusions of this article will be made available by the authors, without undue reservation.

## Ethics statement

All enrolled patients provided written informed consent, in accordance with the principles of the Declaration of Helsinki. Each participating center received the approval of the local Ethics Committee (approval no. CESC code: 4930/AO/2. URC: AOP2073), as well as the written informed consent for the anonymous use of personal data from every patient, in compliance with Italian Legislative Decree 196/2003.

## Author contributions

GS and AH researched data for the article, wrote the article. RR contributed substantially to discussion of the content and reviewed and edited the manuscript before submission. ML, GC, LS, TV, FF, PDS, PS, and AD made substantive intellectual contributions to the study, and reviewed the article. All authors contributed to the article and approved the submitted version.
