# Supplementary material for: The impact of SARS-CoV-2 infection and vaccination on inflammatory arthritis: a cohort study
Source: Front Immunol. 2023 Jul 26;14:1207015. doi: 10.3389/fimmu.2023.1207015 (PMC10410443; doi:10.3389/fimmu.2023.1207015)
Supplement: Supplementary file 1 [file Table_1.docx]

**Supplementary table.** Demographic, clinical aspects, comorbidities and SARS-CoV-2 infection and/or vaccination information collected during the interview

| **Informations collected during interviews** |
| --- |
| 1) Age (years old) |
| 2) Sex |
| 3) Comorbidities: ⋄  • Cardiovascular comorbidities  • Diabetes  • Obesity (BMI > 30.0)  • Pulmonary comorbidities  • Cancer |
| 4) Rheumatic disease activity ^* |
| 5) Ongoing therapy * |
| 6) SARS-CoV-2 infection: ⁺⋄ |
| • If yes: |
| ◦ Date of the positive swab |
| ◦ Date of the negative swab |
| ◦ Symptoms of COVID-19 |
| ◦ Severity of COVID-19 °* |
| ◦ Hospitalization ⋄ |
| ◦ Exacerbation of the joint disease following infection *⋄ |
| ⁃ If yes, |
| - NSAIDs need to control flare up ⋄ |
| - Therapeutic switch following the flare up *⋄ |
| 7) Anti-SARS-CoV-2 vaccination: ⋄ |
| • If not: |
| ◦ Reason for missed vaccination |
| • If yes: |
| ◦ Number of vaccine shots received |
| ◦ Booster shot ⋄ |
| ◦ Date of the last vaccine shot |
| ◦ Type of vaccine received ˇ |
| ◦ Therapy stopped to allow vaccination ⋄ |
| ◦ Side effects within 48 hours of vaccination ⋄ |
| ⁃ If yes: |
| - After what shot |
| - Kind of side effects |
| - Exacerbation of joint disease within 1 month *⋄ |
| ‣ If yes, NSAIDs need to control flare up ⋄ |
| ‣ If yes, therapeutic switch following the flare up *⋄ |

SARS-CoV-2, severe acute respiratory syndrome coronavirus 2; COVID-19, coronavirus disease 19; NSAIDs, non-steroidal anti-inflammatory drugs

^ Disease activity evaluated by ASDAS-CRP (Ankylosing Spondylitis Disease Activity Score-C Reactive protein) and DAS28-CRP (Disease Activity Score-C Reactive Protein), define as remission (ASDAS-CRP <1.3, DAS28-CRP <2.6), low disease activity (ASDAS-CRP 1.3-2.0, DAS28-CRP 2.6-3.2), active disease (ASDAS-CRP ≥2.1, DAS28-CRP ≥3.2).

* Reserved to the physician.

° SARS-CoV-2 severity evaluated by WHO criteria, defined as "asymptomatic or presymptomatic infection [no symptoms that are consistent with COVID-19]; mild illness [any of the various signs and symptoms of COVID-19, e.g., fever, cough, sore throat, malaise, headache, muscle pain, nausea, vomiting, diarrhea, loss of taste and smell but not shortness of breath, dyspnea, or abnormal chest imaging]; moderate illness [evidence of lower respiratory disease during clinical assessment or imaging and oxygen saturation measured by pulse oximetry (SpO2) ≥94% on room air at sea level]; severe illness [SpO2 <94% on room air at sea level, ratio of arterial partial pressure of oxygen to fraction of inspired oxygen (PaO2/FiO2) <300 mm Hg, respiratory rate >30 breaths/min, or lung infiltrates >50%], critical illness [respiratory failure, septic shock, and/or multiple organ dysfunction]".

⋄ "Yes" or "no" responses.

⁺ SARS-CoV-2 infection was considered only if documented in accordance with the legislation in force in Italy, first only with rino/oro-pharyngeal swab for molecular test, then also with rino/oro-pharyngeal swab for rapid antigen test.

ˇ Comirnaty (Pfizer/BioNTech), Spikevax (Moderna), Vaxzevria (Oxford/Astrazeneca).
